# Supplementary material for: Emergent Orientation Selectivity from Random Networks in Mouse Visual Cortex
Source: Cell Rep. Author manuscript; Available in PMC 2018 Oct 10. (PMC6179374; doi:10.1016/j.celrep.2018.07.054)
Supplement: 1 [file NIHMS1505382-supplement-1.pdf]

Cell Reports, Volume *24*

## Supplemental Information

# Emergent Orientation Selectivity

# from Random Networks in Mouse Visual Cortex

**Jagruti J. Pattadkal, German Mato, Carl van Vreeswijk, Nicholas J. Priebe, and David Hansel**

**Supplementary Figure 1**

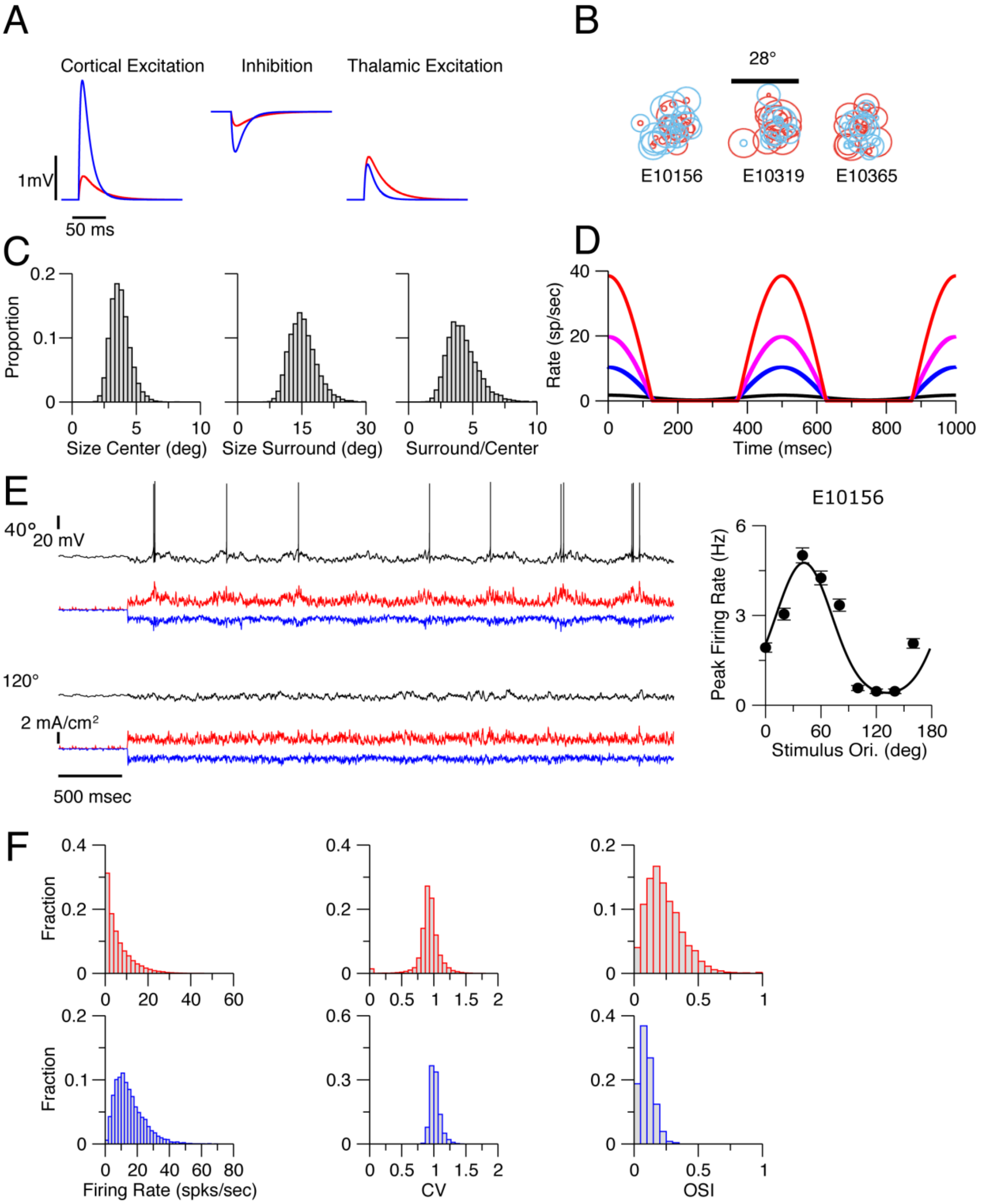


**Figure S1: The large scale spiking model of V1 in the balanced regime.** Related to Figures 2 and 3. Parameters are as in Tables S1 and S2. **A.** PSPs for the cortical excitation (left), the cortical inhibition (middle) and the thalamic excitation (right). Red: PSPs for excitatory postsynaptic neuron. Blue: PSPs for inhibitory post-synaptic neuron. **B.** Receptive fields of thalamic neurons presynaptic to three representative cortical neurons. Thalamic neurons have circular receptive fields modeled as a difference of Gaussian describing OFF and ON subfields (see Methods). Red: ON subfield. Blue: OFF subfield. The radii of the plotted circles are the standard deviation (SD) of the corresponding Gaussians. **C.** Histograms of the radii of center (left) and surround subfields (middle). Right: histogram for the ratio of the surround and the center subfields radii. **D** Responses of LGN neurons are strongly rectified unless the contrast is very low. Firing rate of one typical LGN neuron as a function of time when the spatial frequency of the stimulus is 0.04 cpd, for 4 values of the contrast: $\varepsilon$=100% (red), 50% (magenta), 25% (blue) and 2% (black). The RF is circular with $\sigma_{cx}=\sigma_{cy}=1$ deg , $\sigma_{sx}=\sigma_{sy}=6.3$ deg and $\beta=2.1$ **E.** Voltage traces for one excitatory neuron. Stimulus begins at t=500msec. The drifting grating (SF = 0.03 cyc/deg) is presented at two orientations: 40° (upper trace) and 120° (lower trace). The panels below the voltage traces depict the excitation/inhibition balance. The excitatory (red), inhibitory (blue) currents to the neuron are plotted (note that in simulations all these currents and the voltage are always simultaneously known). Right panel: The tuning curve of the neuron. Average firing rate averaged over 80 sec. Parameters are as in Tables 1 and 2. **F.** Histograms of the peak firing rate (left), the coefficient of variation (CV) of the interspike distribution (middle).

The stimulus is at 0° (SF=0.03 cyc/deg). Histograms are essentially the same for all stimulus orientations. The heterogeneity in firing rates and high temporal variability (CV around 1) of the neurons' discharges are hallmarks of the balanced state. Right panel: The OSI of the peak response (SF=0.03 cyc/deg). Red: Excitatory neurons. Blue: Inhibitory neurons. Inhibitory neurons are much less selective than excitatory neurons in agreement with experimental results (Kuhlman et al., 2011).

**Supplementary Figure 2**

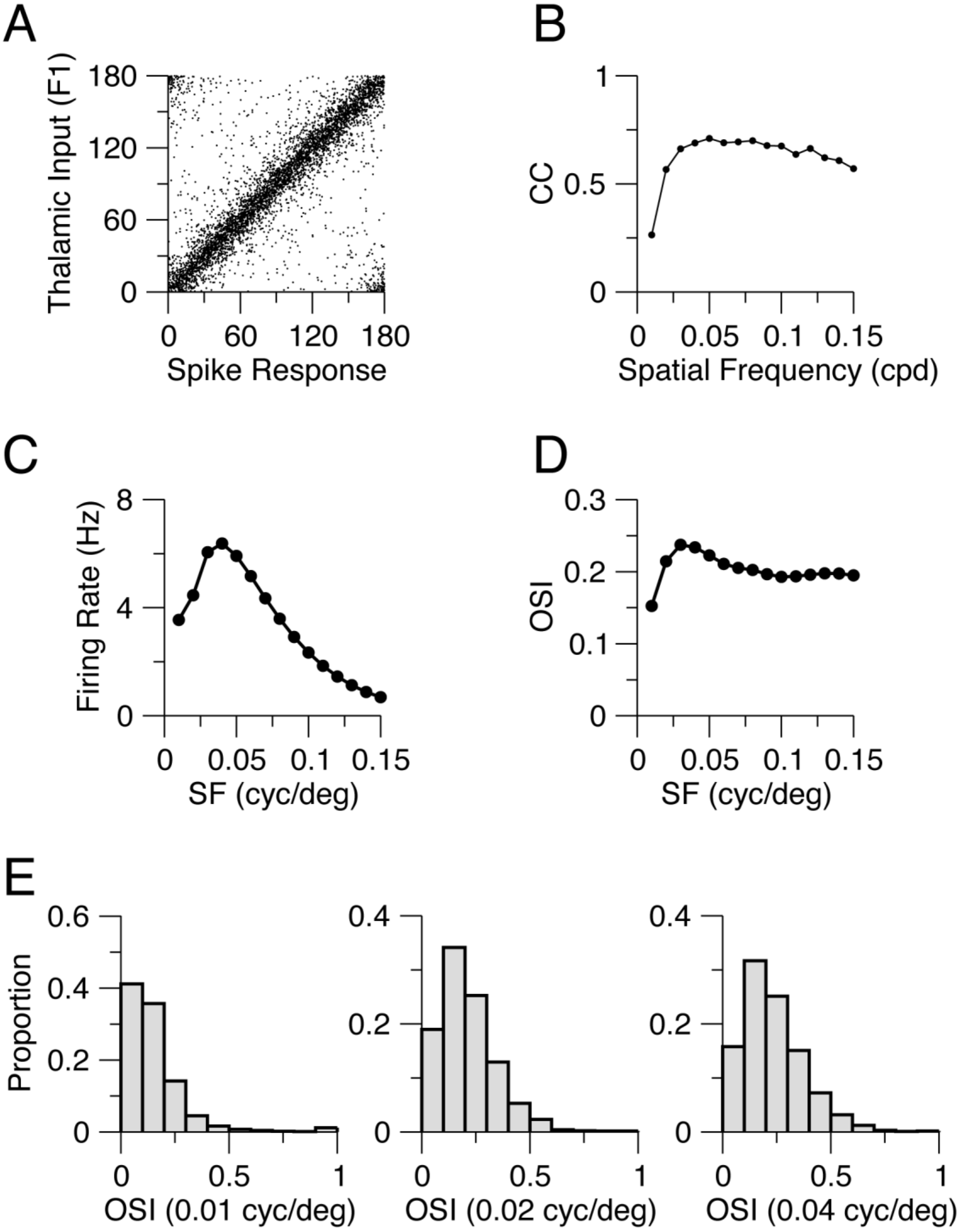


**Figure S2: Dependence of the firing rate and selectivity on spatial frequency in the V1 network model.** Related to Figures 2 and 3. **A.** Preferred orientation of the thalamic excitation vs. preferred orientation of the spike response of the cortical neurons for SF=0.03 cyc/deg (n=5041). **B.** The circular correlation (CC, see Methods) of the thalamic input and spike response preferred orientations vs. the spatial frequency. The dependence on spatial frequency is weak except for low spatial frequency. **C.** Population average of the peak firing rate at preferred orientation (E neurons) vs. grating spatial frequency (See Methods). **D.** Population average OSI of the peak spike response vs. spatial frequency. **E.** Histograms of the OSI for different spatial frequencies. (For 0.03 cyc/deg, see Fig. 2). The histograms are similar except for SF=0.01 cyc/deg.

**Supplementary Figure 3**

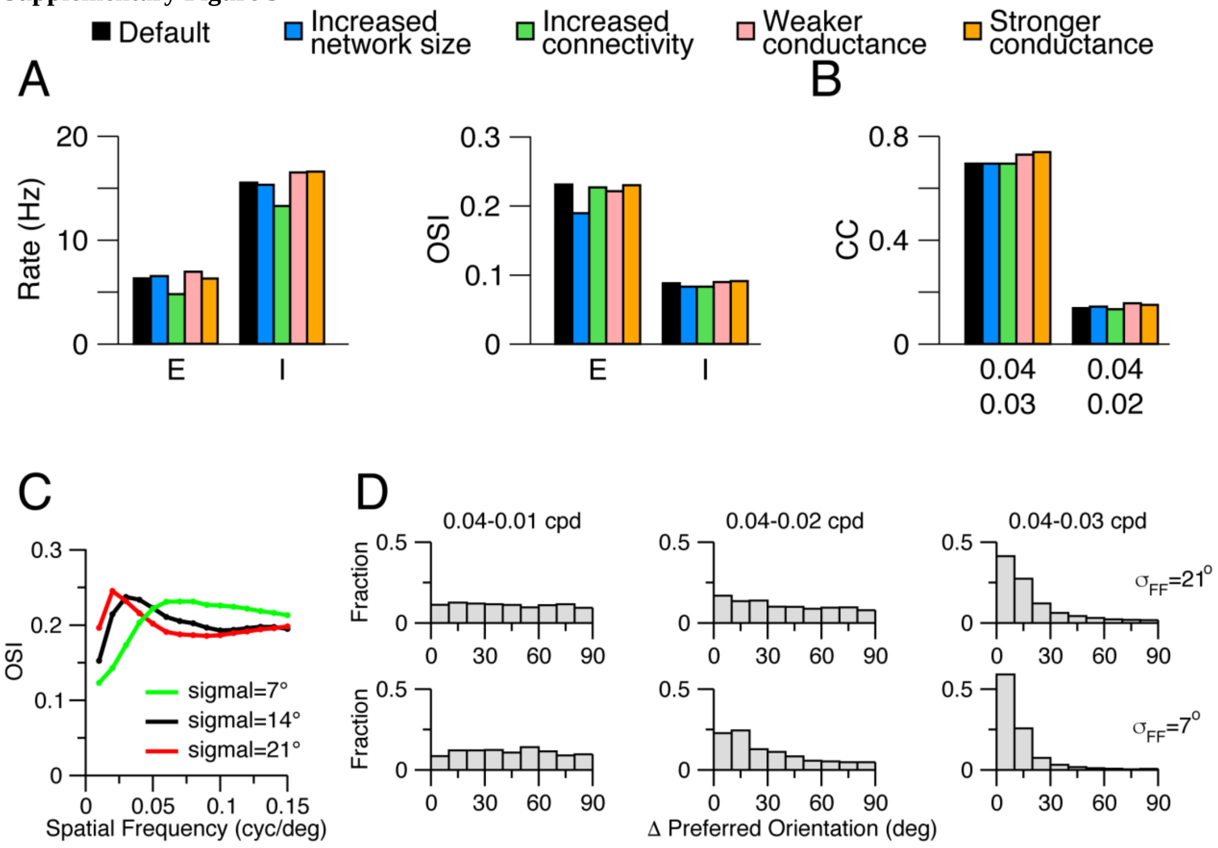


**Figure S3: Robustness to changes in the model network size, connectivity parameters and thalamo-cortical dispersion**. Related to Figures 2 and 3. **A**. Bar charts for the population averaged peak response (orientation of the stimulus: 0°; SF=0.04 cycle/deg) for excitatory (E) and inhibitory (I) neurons (left) and population averaged OSI (right). Black: Default set of parameters (Table 1,2). Blue: $N_E$=78400, $N_I$=19600, $N_L$=40000; other parameters as in Table 1, 2. Green: $K_{EL}$=100; $g_{EL}$ was increased to keep in both populations the firing rate approximately the same.: $g_{EL}$=0.009 mS msec/cm$^2$; Pink: All conductances are multiplied by 0.75; Orange: All conductances are multiplied by 1.25. Other parameters as in Table 1, 2. **B.** Correlation coefficient (CC) for excitatory population between the preferred orientation of spike response for SF=0.04 cyc/deg and SF=0.03 cyc/deg (left) and SF=0.02 cyc/deg (right). For SF=0.01 cyc/deg: CC=0. Color code as in A. **C.** Population average OSI of the peak spike response of excitatory neurons vs. spatial frequency for $\sigma_{FF}$. = 14° (black), 21° (red) and 7° (green). Other parameters as in Table 1, 2. **D.** Histograms of the difference in orientation preference between 0.04 cyc/deg and 0.01 (left), 0.02 (middle) and 0.03 (right) cyc/deg. $\sigma_{FF}$=7° are plotted in the top row. The bottom row shows the histograms for $\sigma_{FF}$ =21°. Compare with the corresponding histograms for $\sigma_{FF}$=14° (Fig. 4). Only neurons with OSI larger than 0.2 for each pair of spatial frequencies are included. For $\sigma_{FF}$=7°: CCs are 0.72 (0.04-0.03 cyc/deg), 0.18 (0.04-0.02 cyc/deg) and 0 (0.04-0.02 cyc/deg). For $\sigma_{FF}$=21°: CC=0.48, (0.04-0.03 cyc/deg), 0.03, (0.04-0.02 cyc/deg), 0 (0.04-0.01 cyc/deg).

**Supplementary Figure 4**

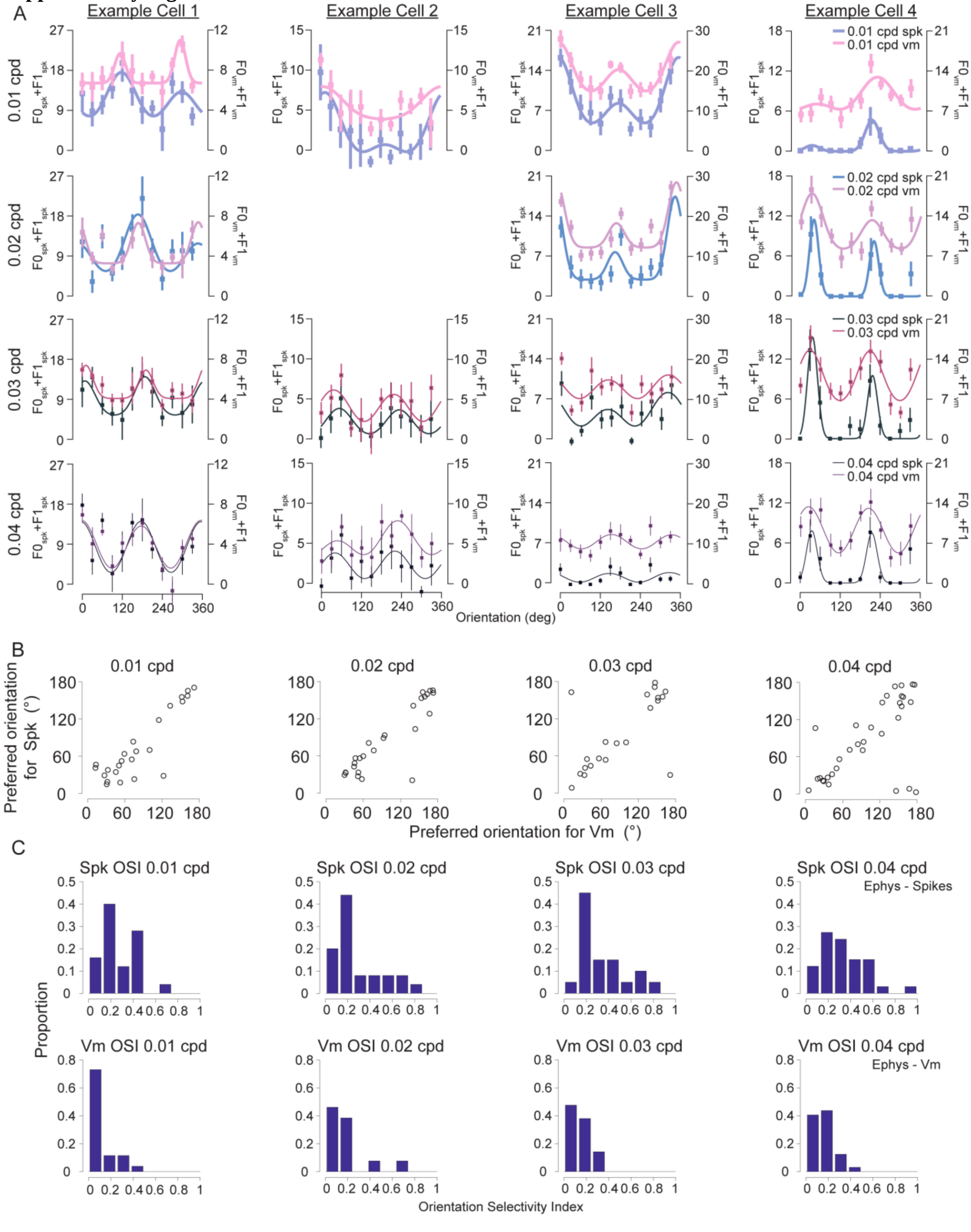


**Figure S4: Vm and spike orientation preferences show similar dependency on spatial frequency in mouse V1.** Related to Figure 4. **A.** Each column represents a neuron. Each row is tuning curves for a different SF. Both the Vm and spikes based tuning curves show similar preference. **B.** The preferred orientation based on spikes vs. the preferred orientation based on Vm for all neurons. **C.** The orientation selectivity index (Methods) for spike rate (top row) and membrane potential (bottom row).

**Supplementary Figure 5**

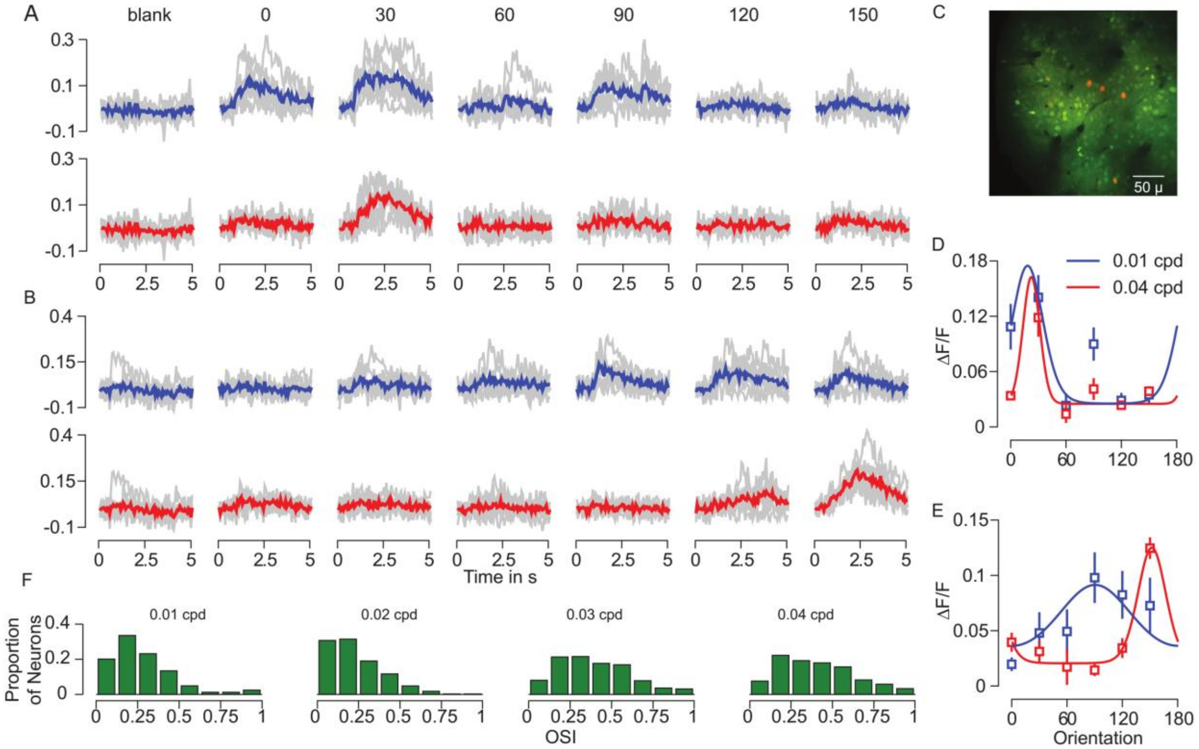


**Figure S5: Example calcium orientation selectivity in mouse V1.** Related to Figure 4. **A, B:** The calcium responses for three example neurons for different orientation conditions and two spatial frequencies (0.01 cpd, blue and 0.04 cpd, red). **C.** Example imaging plane. **D, E:** Orientation tuning curves for different SFs for the neurons represented in A and B. **F.** OSI distribution across all SFs used.

**Supplementary Figure 6**

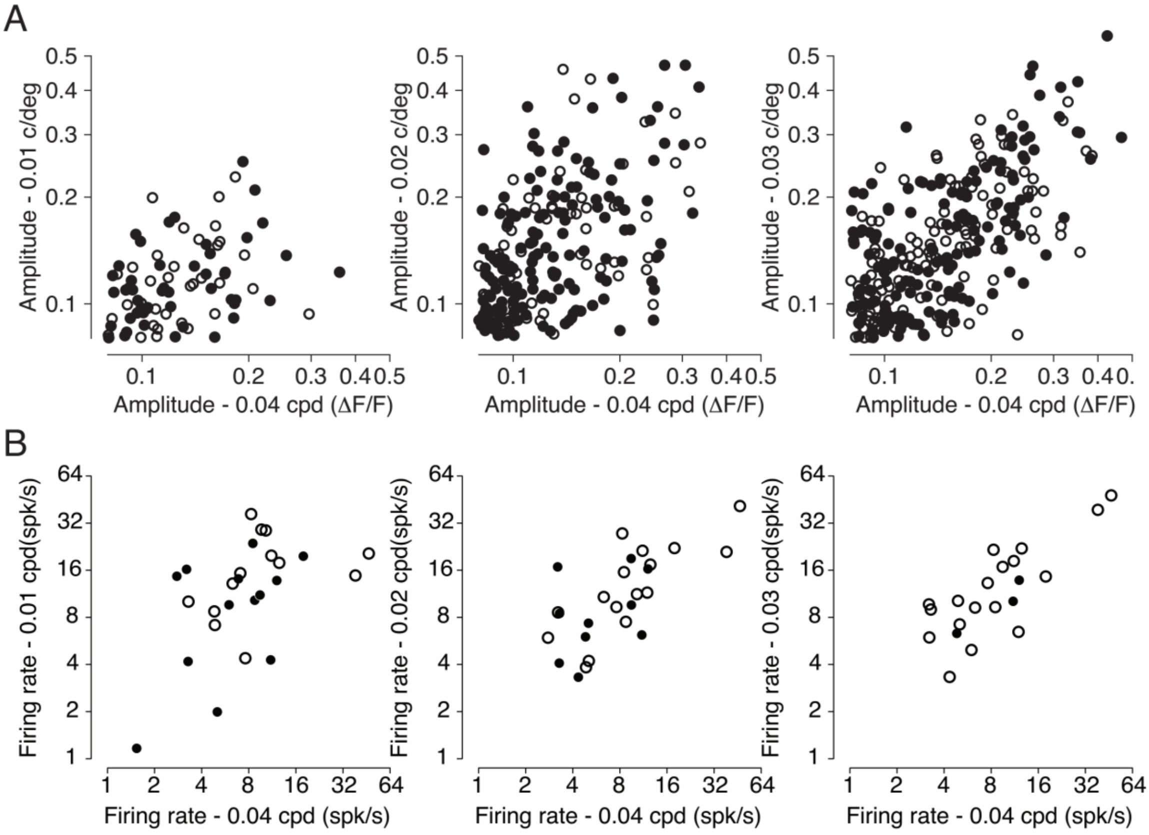


**Figure S6: Response amplitudes across spatial frequencies in mouse V1.** Related to Figure 4**. A:** The amplitude of calcium responses used to extract orientation preference are plotted for 0.01, 0.02 and 0.03 relative to the amplitudes at 0.04 cyc/deg. Closed symbols have significant changes in orientation preference whereas open symbols did not exhibit significant changes in orientation preference. **B:** As in A, but for electrophysiology. Responses had the background response removed by subtraction.

**Supplementary Figure 7**

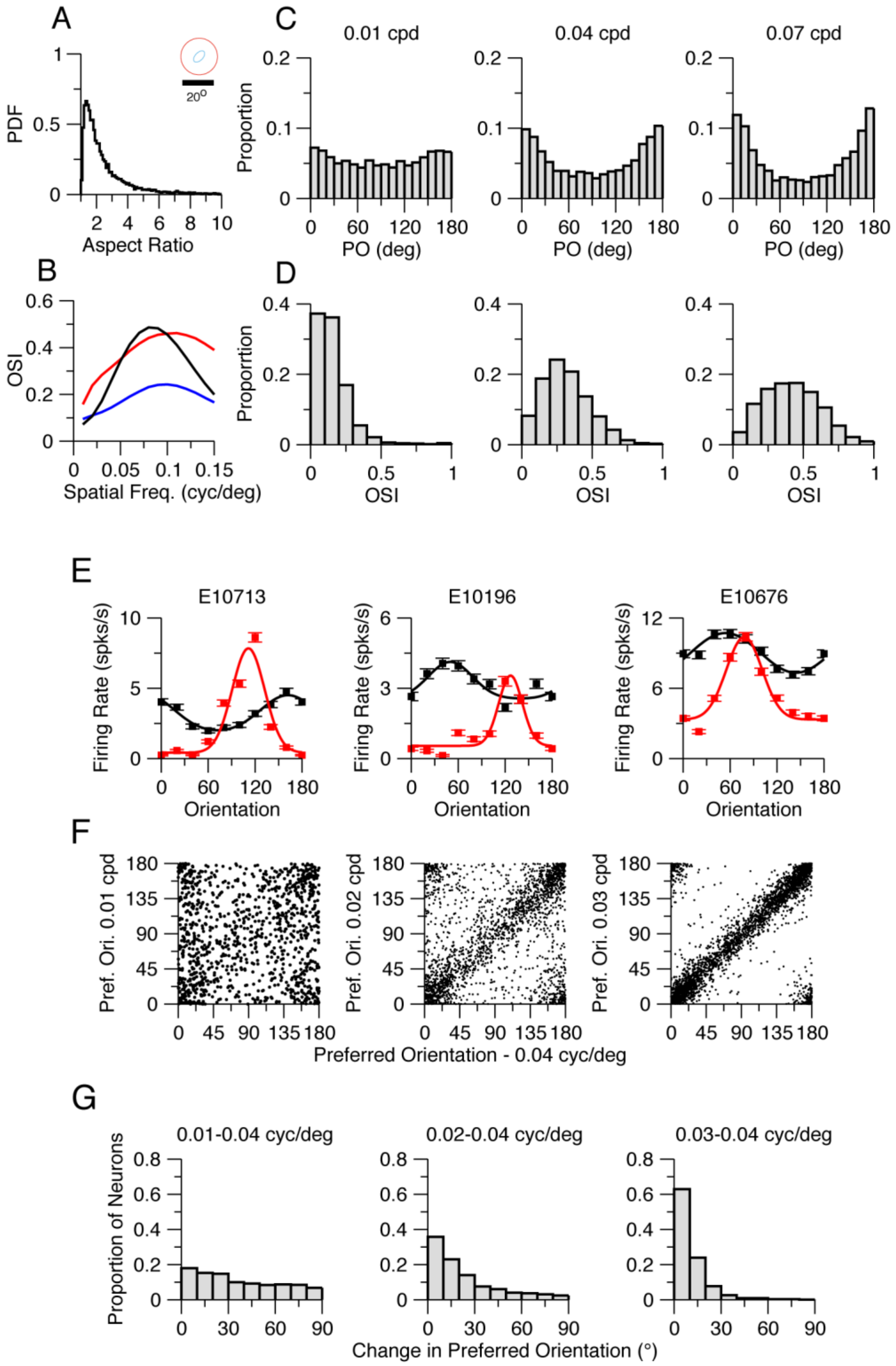


**Figure S7: The V1 network model with elongated thalamic receptive.** Related to Figure 5. Parameters are as in Table S3. **A.** The central subfields of the receptive fields of LGN cells are elongated. Surround subfields are circular. Distribution of the aspect ratio for the center subfield is plotted. Inset: Example of a thalamic neuron receptive field. **B.** Population average OSI for the spike peak response vs. spatial frequency. Red: E cells. Blue: I cells. Black: LGN cells. **C.** Histograms of the preferred orientations of the excitatory neurons for grating with 0.01 (left), 0.04 (middle) and 0.07 (right) cyc/deg. Preferred orientations are bias toward 0° because the distribution of the axis orientation of the center subfield of LGN cells is biased toward this orientation. **D.** Histograms of OSI for E neurons. Left to right: 0.01, 0.04, 0.07 cyc./deg. **E.** Tuning curves of three example E cortical neurons. Black: 0.01 cyc/deg; Red: 0.04 cyc/deg. **F, G.** Population data demonstrating the change in preferred orientation with spatial frequency. CC=0.77 (0.04-0.03 cyc/deg)., 0.33 (0.04-0.02 cyc/deg,) 0.04 (0.04 -0.01 cyc/deg.

**Supplementary Figure 8**

A

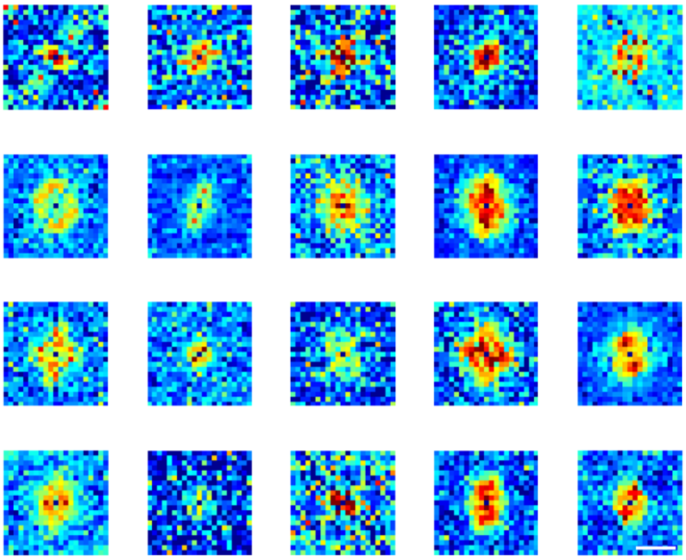

B

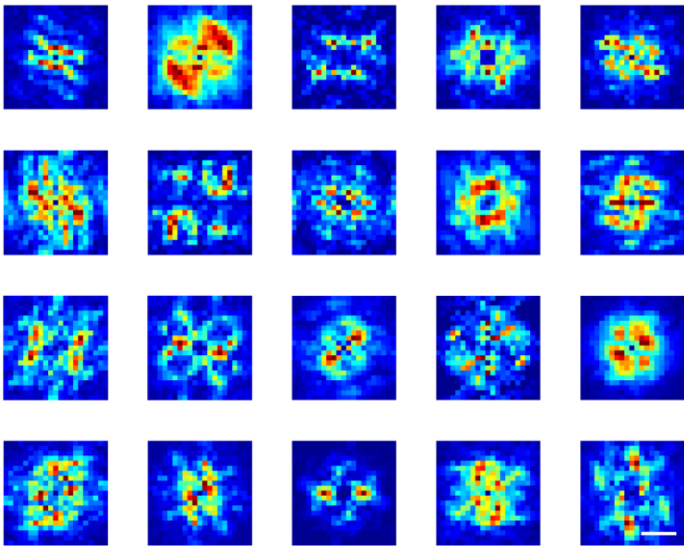

**Figure S8: Examples of Hartley RFs based on electrophysiology and the model.** Related to Figure 4**. A.** 20 example neuron membrane potential responses to the Hartley stimulus in mouse V1. **B**. 20 example responses of neurons to the Hartley stimulus in the V1 network model.

**Supplementary Figure 9**

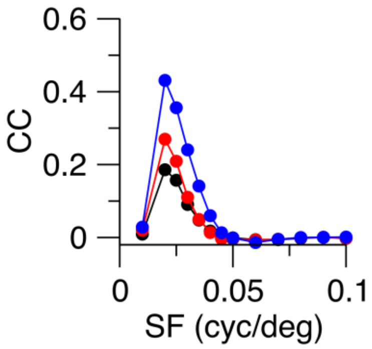


**Figure S9: Correlation coefficient between the orientation of the ON-OFF offset axis and the orientation preference of the thalamic excitation vs. spatial frequency in the model.** Related to Figure 3. Parameters of the model as in the default set. Black: All cells. Red: Offset > 2°. Blue: Offset > 4°.

| | |
|---|---|
| $N_E$ | 32400 |
| $N_I$ | 8100 |
| $K_{AB}$ (A,B=E,I) | 500 |
| $K_{EL}$ | 25 |
| $K_{IL}$ | 100 |
| $g_{EE}$ | 0.011 ms mS/cm$^2$ |
| $g_{IE}$ | 0.067 ms mS/cm$^2$ |
| $g_{EI}$ | 0.029 ms mS/cm$^2$ |
| $g_{II}$ | 0.098 ms mS/cm$^2$ |
| $g_{EL}$ | 0.04 ms mS/cm$^2$ |
| $g_{iL}$ | 0.02 ms mS/cm$^2$ |
| $\tau_{AB}$(A=E,I ; B=E,I,L) | 3 ms |
| $\sigma_{AB}$ (A,B=E,I) | 200 µm |
| $\sigma_{AL}$ (A=E,I) | 100 µm |
| $V_E$ | 0 mV |
| $V_I$ | -80 mV |
| $V_L$ | -65 mV |
| $\rho$ | 0.5 |
| $K_b$ | 500 |
| $g_b$ | 0 |
| $r_0$ | 1 Hz |
| $L_0$ | 0.075 |
| $\varepsilon$ | 1 |

Table S1: Default parameters of the computational model of mouse V1 (Layer 4 and stimulus). Related to model. Related to Figs. 1-6.

| | |
|---|---|
| $N_L$ | 25600 |
| $r_0$ | 1 Hz |
| $\sigma_{cxm}$ (*) | 1.3 |
| $\sigma_{sxm}(*)$ | 2.7 |
| $\beta_m$ | 0.05 |
| $\sigma_{cxs}(*)$ | 0.2 |
| $\sigma_{sxs}(*)$ | 0.2 |
| $\beta_s$ | 0.45 |

Table S2: Parameters of the computational model of mouse V1: default values for the LGN cells. Related to model. Related to Figs. 1-6.

| | |
|---|---|
| $\alpha_m$ | 0 |
| $\alpha_s$ | 0.2 |
| $a_\theta$ | 142 |
| $b_\theta$ | 1128 |
| $c_\theta$ | 21.5 deg |

Table S3: Parameters of the computational model of mouse V1: elongated receptive fields of LGN cells. Related to model. Related to Fig. 5

(*) Parameters of the log-normal distribution are normalized to obtain values in degrees.

DATA AND SOFTWARE AVAILABILITY

Data and software are available upon request. Requests should be sent to the corresponding author.
